# Moral hazard and selection for voluntary deductibles

**DOI:** 10.1002/hec.4134

**Published:** 2020-07-31

**Authors:** Rob J. M. Alessie, Viola Angelini, Jochen O. Mierau, Laura Viluma

**Affiliations:** ^1^ Department of Economics, Econometrics and Finance, Faculty of Economics and Business University of Groningen Groningen The Netherlands; ^2^ Netspar Tilburg The Netherlands; ^3^ Department of Economics VU Amsterdam Amsterdam The Netherlands

**Keywords:** deductible, health insurance, moral hazard, selection

## Abstract

This paper investigates whether the voluntary deductible in the Dutch health insurance system reduces moral hazard or acts only as a cost reduction tool for low‐risk individuals. We use a sample of 14,089 observations, comprising 2,939 individuals over seven waves from the Longitudinal Internet Studies for the Social sciences panel for the analysis. We employ bivariate models that jointly model the choice of a deductible and health care utilization and supplement the identification with an instrumental variable strategy. The results show that the voluntary deductible reduces moral hazard, especially in the decision to visit a doctor (extensive margin) compared with the number of visits (intensive margin). In addition, a robustness test shows that selection on moral hazard is not present in this context.

## 1 INTRODUCTION

It is well‐known that comprehensive (health) insurance may lead to moral hazard—that is, a change in health behavior in response to lower out‐of‐pocket health care costs. Various cost‐sharing options are used by policy makers and health insurers to counteract moral hazard, such as copayments and deductibles. In the managed competition health care system settings of Switzerland, the Netherlands, and (to a lesser extent) the United States, a voluntary deductible is offered to the consumer in addition to a mandatory deductible in return for a premium rebate (van Winssen, Van Kleef, & Van de Ven, 2015; Zweifel & Manning, 2000). However, current empirical evidence on the existence of moral hazard effects in relation to a voluntary deductible in a managed competition setting is mixed. In the Dutch setting, Remmerswaal et al. (2019) compare health care costs before and after the deductible kicks in at age 18 using administrative insurance claims data and show that the voluntary deductible does not reduce the health care costs for this age group. Analyzing the Swiss market using data from the Swiss Health Survey, which covers all of Switzerland, Schellhorn (2001) does not find any significant effects of a deductible on the number of doctor visits. However, Gardiol, Geoffard, and Grandchamp (2005) and Trottmann, Zweifel, and Beck (2012), using data from a large insurance company, and Gerfin and Schellhorn (2006), using data from the Swiss Health Survey, find significant, negative effects of the size of the deductible on health care costs.

Naturally, a degree of selection may occur if the deductible is voluntary. Healthy individuals choose a higher deductible, and less healthy individuals opt for no deductible, which ultimately leads to a transfer of costs from healthy individuals to unhealthy individuals (Nyman, 1999). van Kleef, Beck, van de Ven, and van Vliet (2008), using the data from Switzerland, show that this transfer of costs can be substantial due to a large selection effect. In addition, Croes, Katona, Mikkers, and Shestalova (2018) find substantial selection effects of the voluntary deductible in the Netherlands. Given the mixed evidence related to moral hazard and the high probability of adverse selection, requests in the Dutch press and in politics have been made to abandon the voluntary deductible on the grounds that it undermines the solidarity principle of health insurance and drives up health insurance costs for people who use a lot of care (de Koning & Don, 2019; NPO, 2017).

To provide additional background on this debate, we use panel data from the Longitudinal Internet Studies for the Social sciences (LISS) for the years 2009–2016 to investigate whether the voluntary deductible in the Netherlands has served its purpose of reducing moral hazard or has acted only as a cost reduction tool for low‐risk individuals.

As Einav and Finkelstein (2018) argue, in the absence of randomized trial data (such as the RAND experiment; see, e.g., Aron‐Dine, Einav, & Finkelstein, 2013), credible reduced‐form empirical studies, which often rely on few modeling assumptions, are a powerful tool for retrospectively answering the question whether moral hazard exists. However, several issues complicate the empirical separation of moral hazard and selection effects, which we address in this paper. First, a well‐known methodological issue in this type of research is establishing causality. It is commonly observed that individuals who opt for a deductible use less health care. However, it is unclear whether and how much of this relationship is due to the incentive effect known as moral hazard on the one hand or self‐selection of healthier individuals into insurance contracts with a voluntary deductible on the other hand. From an econometric perspective, even after health status and other individual characteristics are controlled for, some unobserved characteristics may be correlated with both the deductible decision and health care utilization (Finkelstein, 2014). Most of the literature on the effects of insurance coverage that uses observational data solves this problem with various bivariate parametric models that jointly model the selection decision and the health care utilization outcome (e.g., Bolhaar, Lindeboom, & Van Der Klaauw, 2012; Cameron, Trivedi, Milne, & Piggott, 1988; Jones, Koolman, & Van Doorslaer, 2006; Kiil & Arendt, 2017; Schellhorn, 2001). Although bivariate models are identified by functional form due to nonlinearity, most studies supplement the identification strategy using an instrumental variable for insurance coverage (Bratti & Miranda, 2011). Following the literature, we also estimate bivariate models for selection into the deductible and health care utilization using an instrumental variable for the endogenous voluntary deductible. Similar to Schellhorn (2001), we use the availability of supplementary insurance coverage as an instrument because, in the Netherlands, supplementary insurance covers health services that are not covered by basic health insurance, but it does not directly affect treatment choices or out‐of‐pocket payments within the services covered by basic insurance. Moreover, choice of supplementary insurance is strongly correlated with choice of the deductible.

The second econometric issue in our analysis is related to the type of health care utilization measures that we use. We consider four measures in our analysis: visits to a specialist, visits to a general practitioner (GP), visits to a mental health care provider, and days spent in the hospital. Notably, mental health care utilization has not been analyzed before in the context of voluntary deductibles (see Gardiol et al., 2005; Gerfin & Schellhorn, 2006; Schellhorn, 2001). The distribution of these outcome variables includes a large number of individuals with zero visits or days in the hospital, whereas the rest are distributed continuously. Because of this setup, in our analysis, we model both the probability of visiting a doctor or spending time in the hospital and the actual number of visits/days. We show that it is important to allow for both relationships because the moral hazard effects differ between the two types of decisions.

Third, the analysis of moral hazard is made even more complex by the multidimensional nature of the selection effects. Textbook models predict that when there is asymmetric information between the insurance company and customers, individuals with high risks and high expected health care utilization buy health insurance with more coverage, which results in adverse selection (Rothschild & Stiglitz, 1976). The literature contains ample evidence for adverse selection (e.g., Bolhaar et al., 2012; Cameron et al., 1988; Jones et al., 2006). However, some evidence also points to the existence of advantageous selection (Bolhaar et al., 2012; Cutler, Finkelstein, & McGarry, 2008; Finkelstein & McGarry, 2006). Advantageous selection happens when risk is negatively related to other factors that positively influence the demand for insurance. To examine the presence of such factors, we employ a test for multidimensional asymmetric information, as suggested by Finkelstein and McGarry (2006), and provide evidence of adverse selection for the utilization of physical health care and advantageous selection for the utilization of mental health care.

Finally, Einav, Finkelstein, Ryan, Schrimpf, and Cullen (2013) show that in addition to classical adverse selection, in which individuals with characteristics that lead to higher health care utilization select into insurance plans with better coverage, selection on moral hazard may also exist. Selection on moral hazard is the result of individual heterogeneity in the behavioral response to health insurance. In particular, individuals might buy insurance because they expect an increase in their health care consumption due to better coverage. Selection on moral hazard can be particularly relevant for changes in insurance contracts. For example, if an insurer provides a contract with better coverage and relies on average estimates of the price elasticity of demand, the insurer will underestimate the increase in costs due to moral hazard. The more comprehensive coverage will attract individuals whose health care consumption would increase more. On the contrary, if the insurer introduces a deductible to limit health care utilization, the insurer will overestimate the effect of such a decision; that is, higher deductibles will first attract individuals who are less sensitive to health care prices. In this paper, we test for the existence of selection on moral hazard by using models that allow for heterogeneous treatment effects.

As we mentioned previously, in our analysis, we use data from the LISS panel for the years 2009–2016. The panel is based on a true probability sample of Dutch households drawn from the population register by Statistics Netherlands and comprises 2,939 individuals. An advantage of the survey data is that it contains rich sociodemographic, economic, and subjective health information that is not typically observed by the insurer in the Netherlands but can drive consumers' choices. Moreover, this data set enables us to directly control for individual risk aversion, which, according to economic theory, is an important determinant of demand for insurance. In addition, the panel structure of the data allows us to avoid reverse causality issues because we can use independent variables that are determined before the period of health care utilization.

Our contribution to the literature is twofold. First, we show the causal effect of a voluntary deductible on health care utilization and demonstrate the difference in the effects on the extensive versus intensive margin of health care utilization. Second, we add to the scarce evidence on the existence of selection on moral hazard. While we study these issues in the institutional setting of the Dutch health care system, the results may also interest policy makers in other countries that have mandatory private health insurance and voluntary deductibles, such as Switzerland, Germany, and the United States.

The rest of this paper proceeds as follows: Section 2 discusses the regulatory framework and the Dutch health insurance system. Section 3 introduces the theoretical concepts of asymmetric information, adverse and advantageous selection, and moral hazard. Section 4 describes the data, and Section 5 discusses the methodological issues. In Section 6, we present our results, and in the final section, we offer some conclusions.

## 2 REGULATORY FRAMEWORK

We use data from the Netherlands to examine the interdependence between moral hazard and adverse selection. In the Netherlands, insurance companies operate under the Health Insurance Act, introduced in 2006 as a step toward regulated competition. This law requires individuals to take out basic health insurance from a private health insurer. Health insurers are obligated to accept everybody for basic insurance, and premiums are based on community rating (e.g., the insurance premium is the same for all individuals regardless of their health status and other characteristics). Insurers can compete on price and selective contracting. In 2018, individuals could choose among 24 health insurers and 55 different plans for their basic health insurance (Romp & Nijhof, 2018). In reality, however, competition in the market is limited because all the health insurers fall under one of the 10 insurance concerns, four of which insure 86.5% of the population.

The basic health insurance covers a predefined benefits package, and the GP acts as a gatekeeper for specialist care. With a referral from the GP, all medically necessary curative specialist care, including mental health care and hospital care, is covered by basic health insurance. Long‐term care is not covered by basic health insurance but rather by the Long‐term Care Act (Wlz). Supplementary insurance can be purchased to cover services that are not covered by basic health insurance, such as dental care, physical therapy, eyeglasses, plastic surgery, and so forth.

Adults are required to pay a mandatory deductible of €385 (2018) per year from which GP care, obstetric care, and maternity care are exempted. (The mandatory deductible size has increased over years: 2009 = €155; 2010 = €165; 2011 = €170; 2012 = €220; 2013 = €350; 2014 = €360; 2015 = €375; and 2016, 2017, 2018, and 2019 = €385.) In addition to the mandatory deductible, individual adults can opt for a voluntary deductible of €100, €200, €300, €400, or €500 per individual per year.

The law states that the offered premium rebate must be equal for each insured person with the same deductible level within the same health insurance product. In 2014, the premium rebate for the highest deductible level (€500) varied between insurance policies, from €180 to €300 per person per year, the average being €240 per person per year. According to van Winssen et al. (2015), 50% of the Dutch population would profit from a maximum deductible, but only 11% actually had it in 2014. Although the law does not require insurers to exclude the same health care services (i.e., GP care, obstetric, and maternity care) from the voluntary deductible as from the mandatory deductible, all insurers do this.

In addition, all insurers contribute to a risk equalization fund and are compensated for the risk profile of their insured customers from that fund. Under perfect risk equalization, insurers would have perfect knowledge of the risk profile of their insured customers and would be compensated accordingly. In reality, however, risk equalization is not perfect, so insurers can offer large premium rebates in exchange for a higher deductible as a self‐selection mechanism (van Kleef, Van Vliet, & Van de Ven, 2013).

## 3 THEORETICAL FRAMEWORK

### 3.1 Asymmetric information and adverse selection

Loosely following the intuition of Finkelstein and McGarry (2006), we consider a simplified version of the Dutch insurance market in which two insurance policies are available: one with a voluntary deductible (low coverage) and one without a voluntary deductible (high coverage). Individuals can differ on two dimensions: their risk type and their risk preferences (risk aversion). Because the insurer is not allowed to risk‐rate the premiums, individuals possess private information about their risk type and risk preferences. In a one‐dimensional setting, high‐risk individuals would buy high‐coverage insurance and low‐risk individuals would buy low‐coverage insurance, resulting in a correlation between coverage and health care utilization, which is referred to as ‘adverse selection’ (Zweifel & Manning, 2000). The existence of such a correlation has been used as evidence of asymmetric information (Chiappori & Salanie, 2000). In the case of multidimensional heterogeneity, this correlation might not exist, even in the presence of asymmetric information. If risk aversion also affects the choice of insurance coverage, more risk‐averse people would buy high‐coverage insurance, and less risk‐averse people would buy low‐coverage insurance. Combining these two dimensions can yield unpredictable results. For example, if high‐risk individuals are, on average, less risk averse than low‐risk individuals, we might observe zero correlation between insurance coverage and health care utilization. Moreover, if the difference in risk aversion is large enough, this might even result in a negative correlation, indicating ‘advantageous selection,’ as shown by Bolhaar et al. (2012). This idea is easy to extend to a model with more than two dimensions of individual heterogeneity.

Finkelstein and McGarry (2006) propose a simple test for multidimensional asymmetric information. They argue that any available information that is not used by the insurer can indicate asymmetric information if it correlates with both insurance coverage and health care utilization. If an individual characteristic has similar signs for both correlations, there should be adverse selection and/or moral hazard, whereas opposite signs indicate the presence of advantageous selection. We employ this method to test for asymmetric information in Section 6.2. In addition, we investigate the existence of a selection effect by testing whether healthier and less risk‐averse people are more likely to opt for a voluntary deductible.

### 3.2 Moral hazard

Moral hazard is typically defined as excess demand for health investments due to having health insurance (Pauly, 1968). This leads policy makers to introduce mechanisms that reduce moral hazard in medical care consumption, such as copayments or deductibles.

The uptake of health insurance lowers the price of health care and reduces other consumption by the insurance premium. This means that households with (more) insurance will maintain a higher health level than those without insurance. In addition, insurance creates an ex post transfer of income from the healthy to the infirm, and this may increase utilization through an income effect on the demand for health care; in addition, it offers access to expensive health care that would not otherwise be affordable (Nyman, 1999; Nyman & Maude‐Griffin, 2001; Pauly, 1968). Therefore, individuals who choose less insurance coverage (or a higher voluntary deductible) and experience a negative health shock consume less health care than they would without the voluntary deductible (for a detailed discussion, see Bolhaar et al., 2012).

In addition, Einav et al. (2013) show that individual heterogeneity in the behavioral response to health insurance can lead to selection on moral hazard. In particular, individuals might choose the size of the deductible expecting an increase in their health care consumption due to better coverage.

In our setting, we investigate moral hazard by testing whether people who opt for a deductible use less health care given their health status. To allow for endogenous selection into the voluntary deductible, in our empirical approach, we employ a linear regression with endogenous treatment and an exclusion restriction. In addition, we test for selection on moral hazard by allowing for selection on returns in our empirical models.

## 4 DATA

For the purpose of this study, we use data from the LISS panel. The panel is based on a true probability sample of Dutch households drawn from the population register by Statistics Netherlands. It consists of 4,500 households and comprises approximately 8,000 individuals. Households that could not otherwise participate are provided with a computer and Internet connection. In terms of observable background characteristics, the LISS panel is representative of the Dutch population.

The core study of the LISS panel contains several questionnaires, each administered on a yearly basis. This paper combines information from the Health study and the study Measuring Higher Order Risk Attitudes of the General Population (hereinafter, ‘Risk Attitudes’). The Health questionnaire contains information on participants' medical history, lifestyle, health care utilization, and health insurance information. The socioeconomic and demographic information is updated each month. In addition, we include a risk aversion measure from the Risk Attitudes study.

For our analysis, we pool seven waves of the Health section of the LISS panel. These cover the years 2009–2016, with the exception of 2014 when the Health questionnaire was not administered. In the waves before 2009, the questions related to health insurance in the Health questionnaire were formulated differently and therefore are not useful for our analysis. This process yields 38,551 observations from 9,638 individuals for us to start with. The Risk Attitudes questionnaire was administered to a random sample of 3,457 members of the LISS panel in 2009. We assume that risk aversion remains constant during the time period covered by our data and assign this risk attitude information to each panel member in each wave they participated in for the Health study. We drop observations with missing values in our variables of interest and exclude all participants younger than 18 years of age because children do not have a deductible in the Dutch health insurance system. This approach leaves us with a final sample of 14,089 observations from 2,939 individuals over seven waves.

### 4.1 Health care utilization

We use four different types of health care as outcome variables from the LISS data. As a measure of primary health care utilization, we use the yearly number of visits to a GP. Primary health care is usually not included in the mandatory or voluntary deductibles in the Netherlands, but a referral from a GP is necessary to access secondary health care, which is included in the deductible. As a measure of secondary health care utilization, we use the yearly number of visits to a medical specialist. The tertiary health care utilization measure is the number of days spent in a hospital during last hospitalization if hospitalized within the last 12 months (this variable is zero if a person has not been hospitalized). Finally, because mental health and mental health care often follow different patterns than physical health and physical health care (see, e.g., Blanchflower & Oswald, 2017, Bolhaar et al., 2012), we include a separate measure of the number of visits to mental health care providers in the last 12 months.

All four of our health care utilization measures suffer from the common self‐reporting problems. However, according to a study by Dalziel, Li, Scott, and Clarke (2018), the recall period of 12 months seems to produce the least biased health care utilization numbers compared with other recall periods, and the bias is more likely to be downward.

### 4.2 Voluntary deductible

Our main variable of interest is a binary variable indicating whether a person opted for a voluntary deductible. Initially, 12.25% of our sample did not answer the question about the voluntary deductible or answered ‘do not know.’ However, we exploit two characteristics of the Dutch insurance market to reduce the number of missing values. First, the default choice in any insurance contract is to have no voluntary deductible. Thus, a person needs to actively opt‐in for the voluntary deductible and also choose the size of the deductible. Therefore, we reason that people who do not know if they have a voluntary deductible most likely do not have it. Second, another option when the person does not make an active choice about the voluntary deductible is to let the insurance contract roll over from the previous year with the same conditions. Therefore, for all missing values, we first check if the person had a voluntary deductible during the previous year, and if they did not change their insurer, we impute the previous year's value for voluntary deductible. For participants with a missing value in the previous year, we impute a zero, assuming no voluntary deductible. ^1^As a robustness test, we also perform the main analysis without the imputed data. The results tend to be slightly less precise due to smaller sample sizes but are generally robust to this exclusion. These results are available on request. Table 1 summarizes the proportion of people who opted for a voluntary deductible in each wave. Still, in our sample, the proportion of people with a voluntary deductible is slightly higher than in the total population (see Romp & Merkx, 2016). This suggests that our approach of assigning missing values as having no deductible does not lead to an underestimation of people with a voluntary deductible.

**TABLE 1:** Proportion of individuals with a voluntary deductible

Year	Voluntary deductible (%)	
	No	Yes
2009	87.34	12.66
2010	83.86	16.14
2011	82.56	17.44
2012	80.6	19.4
2013	83.57	16.43
2015	79.93	20.07
2016	81.93	18.07
Total	82.99	17.01

### 4.3 Control variables

Both theory and the literature (e.g., van Winssen, Van Kleef, & Van de Ven, 2016) predict that risk aversion is a potentially important determinant of opting for a voluntary deductible in the Netherlands. The LISS data allow us to model risk aversion explicitly. We include a risk aversion measure from the Risk Attitudes study in our analysis. Following Noussair, Trautmann, and Van de Kuilen (2013), we measure an individual's risk aversion as the number of safe choices he or she made out of the five decisions involving a sure payoff and a risky lottery. The sure payoff in the five games was €20, €25, €30, €35, and €40. The risky option in all cases was an equiprobable lottery to receive either €5 or €65. We assume that risk aversion is constant over the seven waves included in this study.

We model the risk type of participants with lagged health status variables. We argue that lagged health status is more relevant than current health status because, in the Netherlands, insurance decisions are made at the end of the previous year. We include a binary variable indicating ‘good’ or ‘very good’ self‐assessed health as a measure of subjective health. Another binary variable indicates whether a person has any chronic conditions, representing an arguably more objective measure of risk type. Finally, we include a measure of mental health, computed from the five‐question Mental Health Inventory (MHI‐5) questionnaire included in the LISS Health study, which can be used to screen for depressive symptoms and anxiety (Yamazaki, Fukuhara, & Green, 2005). The score for the MHI‐5 is determined by summing the scores of each question item (answers range in the frequency of experiencing a symptom from 1 = all of the time to 6 = never) and then transforming the raw scores to a 0–100‐point scale, where 100 indicates perfect mental health.

Other dimensions of individual heterogeneity are sociodemographic and socioeconomic characteristics. This information about the sample comes from the Background variables of the LISS panel, which are updated every month. Similar to the health status variables, we match each wave of the Health data with the Background variables from November of the prior year when insurance decisions were made. Finally, we include year dummies in all models to control for any policy changes in insurance coverage and any other secular trends. Tables 2 and 3 summarize all the data and their descriptives. In addition, Appendix S1 provides a full list of variables with detailed descriptions.

**TABLE 2:** Descriptive statistics, whole sample, and subsamples with and without a voluntary deductible

Variable	All		With a voluntary deductible		No voluntary deductible		*t* test
	Mean	*SD*	Mean	*SD*	Mean	*SD*	Diff
Specialist visits	1.379	4.778	1.062	6.651	1.444	4.292	0.000
GP visits	2.258	5.108	1.810	4.085	2.350	5.288	0.000
Mental health care visits	0.501	3.887	0.469	3.004	0.508	4.044	0.659
Days in the hospital	0.521	4.264	0.302	2.803	0.565	4.504	0.006
Risk aversion	3.404	1.680	3.221	1.689	3.442	1.676	0.000
Male	0.494	0.500	0.541	0.498	0.484	0.500	0.000
Age	53.05	15.90	48.90	15.04	53.90	15.93	0.000
Educ.low	0.347	0.476	0.263	0.440	0.364	0.481	0.000
Educ.mid	0.324	0.468	0.306	0.461	0.328	0.469	0.042
Educ.high	0.329	0.470	0.431	0.495	0.308	0.462	0.000
Employed	0.522	0.500	0.644	0.479	0.497	0.500	0.000
Married	0.75	0.433	0.724	0.447	0.755	0.430	0.001
Log‐income	7.839	0.504	7.891	0.533	7.828	0.497	0.000
Good health	0.221	0.415	0.313	0.464	0.202	0.401	0.000
Mental health inventory (MHI‐5)	79.45	13.63	79.65	13.49	79.41	13.66	0.620
Chronic cond.	0.332	0.471	0.225	0.418	0.354	0.478	0.000
Smokes	0.192	0.394	0.210	0.408	0.188	0.391	0.018
Observations	14,089		2,396		11,693		

Abbreviation: GP, general practitioner.

**TABLE 3:** Distribution of the outcome variables

Value	Specialist visits		GP visits		Mental health care visits		Days in the hospital	
	Freq.	Cum.%	Freq.	Cum.%	Freq.	Cum.%	Freq.	Cum.%
0	8,377	59.46	4,306	30.56	13,233	93.92	12,583	89.31
1	1,951	73.31	3,140	52.85	127	94.83	538	93.13
2	1,433	83.48	2,275	69.00	99	95.53	299	95.25
3	729	88.65	1,397	78.91	72	96.04	144	96.27
4	540	92.48	1,111	86.80	70	96.54	92	96.93
5	313	94.71	671	91.56	95	97.21	119	97.77
6	227	96.32	441	94.69	65	97.67	52	98.14
7	79	96.88	112	95.49	24	97.84	66	98.61
8	118	97.71	197	96.88	65	98.3	33	98.84
9	25	97.89	26	97.07	12	98.39	12	98.93
10	110	98.67	197	98.47	75	98.92	26	99.11
11	12	98.76	8	98.52	2	98.94	8	99.17
12	35	99.01	78	99.08	40	99.22	26	99.35
13	9	99.07	4	99.11	2	99.23	6	99.40
14	6	99.11	11	99.18	4	99.26	20	99.54
15	31	99.33	31	99.40	13	99.35	3	99.56
16	6	99.38	7	99.45	8	99.41	5	99.60
17	3	99.40	1	99.46	1	99.42	4	99.62
18	4	99.43	2	99.47	2	99.43	3	99.65
19	2	99.44	2	99.49	1	99.44	1	99.65
20	24	99.61	32	99.72	24	99.61	3	99.67
21	2	99.62	0	99.72	1	99.62	10	99.74
22	2	99.64	0	99.72	2	99.63	0	99.74
23	1	99.65	1	99.72	1	99.64	1	99.75
24	2	99.66	4	99.75	11	99.72	0	99.75
25	13	99.75	3	99.77	3	99.74	5	99.79
>25	35	100.00	32	100.00	37	100.00	30	100.00
Total	14,089	100	14,089	100	14,089	100	14,089	100

Abbreviation: GP, general practitioner.

## 5 METHODS

### 5.1 Asymmetric information and selection effects

The aim of this paper is to estimate the adverse (advantageous) selection and moral hazard effects of having a voluntary deductible. In the Dutch institutional setting, insurance companies are not allowed to risk‐rate insurance premiums, which means that the existence of asymmetric information is inevitable. Individuals are free to choose the size of the voluntary deductible, and the insurance company cannot refuse them. Nevertheless, because van Winssen et al. (2015) find that the uptake of a voluntary deductible is much lower than would be profitable, it is an open question whether consumers use their private information. Accordingly, when we refer to ‘asymmetric information,’ we mean the individual's use of their private information in insurance coverage decisions and health care utilization.

We start with a descriptive analysis and establish that differences in health care utilization exist between people with and without a deductible. We perform independent sample *t* tests to compare the two groups. However, as Finkelstein and McGarry (2006) show, the mere existence of a difference might not reveal the full extent of the asymmetric information if there are multiple dimensions of asymmetric information. In that case, as discussed in Section 3, we can reject the null hypothesis of symmetric information if, conditional on the information used by the insurer in setting insurance prices, we observe some other characteristic that is correlated with both the insurance coverage and health care utilization of an individual. Because Dutch insurers are not allowed to risk‐rate premiums, any individual characteristics that are correlated with both insurance coverage and health care utilization would be an indication of asymmetric information. Similar to Bolhaar et al. (2012), to perform this test, we model selection into the deductible with a linear probability model (LPM) ^2^As a robustness check, we also estimate the model with probit regression. The average marginal effects of the probit model are virtually identical to the LPM coefficients. in Equation 1:

(1) $$d_{\mathrm{it}}=x_{\mathrm{it}}^{\prime }\beta +\varepsilon_{\mathrm{it}},$$

where *d*_*it*_ is a binary variable indicating that person *i* has opted for a voluntary deductible in year *t*, ***x***_***it***_ is a (column) vector of individual characteristics that are measured before the decision to take the deductible, ***β*** is a (column) vector of parameters, and *ε*_*it*_ is the error term, which is assumed to be conditionally mean independent of ***x***_***it***_. We estimate the model using ordinary least squares (OLS), and standard errors are clustered at the household level. Any significant coefficient *β*_*k*_ means that the uptake of the deductible is not random, and we can interpret this as an indication of selection effects.

In addition, we model health care utilization with four OLS regressions, as in Equation 2:

(2) $$y_{\mathrm{it}}=x_{\mathrm{it}}^{\prime }\gamma +u_{\mathrm{it}},$$

where *y*_*it*_ is a count variable indicating the number of visits to the specialist/GP/mental health care provider or days in the hospital for person *i* in year *t*, ***γ*** is a vector of parameters, and *u*_*it*_ is the error term. Because of the panel structure of our data, we cluster the standard errors at the household level. In addition, we report the correlation coefficient *ρ* between the residuals ${\hat{\varepsilon }}_{\mathrm{it}}$ and ${\hat{u}}_{\mathrm{it}}\;$ for each of the four dependent variables in Equation 2. Similar to Bolhaar et al. (2012), taking the results of Equations 1 and 2 together, we can deduce asymmetric information. In our case, opposite signs for *β*_*k*_ and *γ*_*k*_ mean that an individual characteristic that is associated with a lower probability of having a deductible increases health care utilization, which suggests adverse selection and/or moral hazard. Accordingly, the same signs for *β*_*k*_ and *γ*_*k*_ would mean advantageous selection.

### 5.2 Moral hazard effects

Having established the nature of the selection effects, we turn to estimating moral hazard effects while controlling for sources of selection: risk type, risk preferences, and socioeconomic status. As we discussed in Section 3, moral hazard is expressed as the effect of having a voluntary deductible on health care utilization, conditional on individual's health status and preferences.

We start with a general model (Equations [3]–[7]) that specifies the two potential levels of health care utilization (*y*_*it*1_, *y*_*it*0_), which are observed if individual *i* in year *t* opts for a voluntary deductible (*d*_*it*_ = 1) or does not (*d*_*it*_ = 0). The potential outcomes of the treatment are assumed to depend linearly on a vector of observable characteristics $x_{\mathrm{it}}^{\prime }$ and unobservables (*u*_*it*1_, *u*_*it*0_). The decision process for the choice of deductible is posed as a function of observables *z*_*it*_ and $x_{\mathrm{it}}^{\prime }$ and unobservables *v*_*it*_, and it is linked to the observed outcome *y*_*it*_ through the latent variable *d*_it_^*^. In the general case, it is assumed that *u*_*it*1_, *u*_*it*0_, and *v*_*it*_ are independent of *z*_*it*_, conditional on $x_{\mathrm{it}}^{\prime }$. In addition, the probability of having a deductible is a function of *z*_*it*_, conditional on $x_{\mathrm{it}}^{\prime }$ (Brave & Walstrum, 2014).

(3) $$y_{\mathrm{it}}=d_{i}y_{\mathrm{it}1}+\left(1-d_{\mathrm{it}}\right)y_{\mathrm{it}0};$$

(4) $$y_{\mathrm{it}1}=x_{\mathrm{it}}^{\prime }\beta_{1}+u_{\mathrm{it}1};$$

(5) $$y_{\mathrm{it}0}=x_{\mathrm{it}}^{\prime }\beta_{0}+u_{\mathrm{it}0};$$

(6) $${d_{\mathrm{it}}}^{*}=x_{\mathrm{it}}^{\prime }\beta_{d}+\gamma z_{\mathrm{it}}-v_{\mathrm{it}};$$

(7) $$d_{\mathrm{it}}=\left\{\begin{array}{c} 1\;\text{if}\;{d_{\mathrm{it}}}^{*}>0\\ \;0\;\text{otherwise}. \end{array}\right)$$

In Equation 6, *z*_*it*_ is an instrumental variable. Even though $x_{\mathrm{it}}^{\prime }\;$ contains a large number of important covariates, such as risk aversion, health status, and socioeconomic status, there may still be some unobserved variables correlated with the voluntary deductible choice and health care utilization variables. The literature has approached the choice of a valid instrumental variable for insurance coverage in various ways. Some studies have used various socioeconomic characteristics as instrumental variables (Buchmueller, Couffinhal, Grignon, & Perronnin, 2004; Harmon & Nolan, 2001; Höfter, 2006), but this approach might not be ideal because these variables can be correlated with health care utilization, as shown by Doorslaer, Koolman, and Jones (2004) and Fletcher and Frisvold (2009). Other researchers, such as Jones et al. (2006) and Bolhaar et al. (2012), use lagged information on access to employer‐provided health care or insurance as an instrumental variable for private health insurance in countries with public health insurance. In the literature that specifically focuses on the effect of deductibles, Schellhorn (2001) employs a bivariate Poisson generalized method of moments approach with endogenous treatment. Schellhorn uses the availability of supplementary insurance cover and three dummies that categorize regions with a similar premium level along with the predicted choice of the deductible as instruments for the deductible choice. Similar to Schellhorn (2001), we argue that in the Netherlands, having supplementary health insurance is exogenous to the utilization of basic health care. The Dutch supplementary insurance covers health services that are not covered by the basic health insurance, such as physiotherapist visits, dental care, eyeglasses, and so forth, but it does not directly affect the treatment choices or out‐of‐pocket payments within the services covered by the basic insurance. Therefore, it should not directly influence the number of visits to physicians. Yet choosing supplementary insurance is strongly correlated with choosing the deductible. Both are influenced by unobservable personal health and risk preferences. Therefore, we employ the availability of supplementary insurance as an instrumental variable for the voluntary deductible. ^3^The panel structure of our data allows creating additional instruments by interacting the supplemental insurance with the year dummies. In a robustness analysis, including these additional instruments in our models does not change the results, hence they are left out of our main analysis. These results are available on request.


We begin our analysis with a restricted case, in which we assume that the effect of the deductible on health care utilization is homogeneous. In our potential outcome setup, this means that ***β***_**1**_ = ***β***_**0**_ and the treatment effect of the deductible is *E*(*y*_*it*1_ − *y*_*it*0_) = *δ* for all *i* and *t.* This setup allows us to model moral hazard by applying bivariate models. Because our health care utilization variables are corner solution responses with a corner at 0 (see the distributions in Table 3), we model both the decision to have any doctor visits (extensive margin) and the number of visits (intensive margin). Accordingly, we employ a bivariate probit model for the choice to have any physician visits and a log‐linear regression with endogenous treatment for the number of visits.

First, we model the choice to have any physician visits. We apply the bivariate probit model to two binary dependent variables, *y*_*it*_, which represents having any physician visits/hospital stays, and *d*_*it*_, which represents having a voluntary deductible. We allow for correlation between the corresponding error terms. Given the observed covariates $x_{\mathrm{it}}^{\prime }$, we can write the bivariate model in terms of a latent variable specification:

(8) $$\left\{\begin{array}{c} y_{\mathrm{it}}^{*}=\delta^{\mathrm{ext}}d_{\mathrm{it}}+x_{\mathrm{it}}^{\prime }\beta +u_{\mathrm{it}}\\ d_{\mathrm{it}}^{*}=\gamma z_{\mathrm{it}}+x_{\mathrm{it}}^{\prime }\beta_{d}-v_{\mathrm{it}}\; \end{array}\right),$$

where *δ*^*ext*^ is the coefficient for the effect of the voluntary deductible on the extensive margin, which in our case can be interpreted as the causal effect of moral hazard. ***β*** and ***β***_***d***_ are vectors of coefficients for the covariates $x_{\mathrm{it}}^{\prime }$, and *z*_*it*_ is the instrument. The observed values are then written as follows:

(9) $$y_{\mathrm{it}}=\left\{\begin{array}{c} \;1\;\text{if}\;y_{\mathrm{it}}^{*}>0\\ \;0\;\text{otherwise} \end{array}\right);$$

(10) $$d_{\mathrm{it}}=\left\{\begin{array}{c} \;1\;\text{if}\;d_{\mathrm{it}}^{*}>0\\ \;0\;\text{otherwise} \end{array}\right).$$

We assume that the error terms are drawn from a standard bivariate normal distribution with zero means, unit variances, and correlation coefficient *ρ*:

(11) uitvit~N001ρρ1.

Assuming that the model is correctly specified, *ρ* = 0 implies that *d*_*it*_ is exogenous with respect to *y*_*it*_. Consistent estimation of the unknown parameters in the model can be achieved using the method of maximum likelihood. Given our data structure, we cluster the standard errors at the household level.

To model the number of visits, we apply a log‐linear regression with endogenous treatment to the observations, where *y*_*it*_ > 0. The model is composed of an equation for the outcome *y*_*it*_ and an equation for the endogenous treatment *d*_*it*_. The endogenous variable *d*_*it*_ for voluntary deductible is modeled as in Equations 4 and 6. The health care utilization *y*_*it*_ is a linear model:

(12) $$\ln\left(y_{\mathrm{it}}\right)=\delta^{\mathrm{int}}d_{\mathrm{it}}+x_{\mathrm{it}}^{\prime }\gamma_{1}+u_{\mathrm{it}}.$$

The error terms are drawn from a standard bivariate normal distribution with zero means, unit variances, and correlation coefficient *ρ*, the same as in Equation 11. We interpret *δ*^*int*^ as the causal effect of moral hazard on the intensive margin. We estimate the model with quasi‐maximum likelihood, and we cluster standard errors at the household level.

Both *δ*^*ext*^ and *δ*^*int*^ show the incentive effect of a voluntary deductible conditional on individual characteristics. According to theory, when facing the same health shock, people with a voluntary deductible would use less health care than those without a voluntary deductible. Accordingly, we expect negative signs for *δ*^*ext*^ and *δ*^*int*^.

In the next step, we relax the homogeneity assumption. As Einav et al. (2013) demonstrate, individuals might buy insurance because they expect an increase in their health care consumption due to better coverage. Einav et al. (2013) call this phenomenon ‘selection on moral hazard.’ In the econometrics literature, it is more generally known as ‘selection on returns’ or ‘essential heterogeneity.’ Essential heterogeneity arises when individuals decide to take the treatment in relation to their expected response to the treatment, meaning that there is individual heterogeneity in treatment effects (Brave & Walstrum, 2014).

Marginal treatment effect (MTE) estimators have been developed to study the impact of a treatment that is likely to vary within a population in correlation with observed and unobserved characteristics, for example, when individuals self‐select into a treatment. The MTE concept was defined by Björklund and Moffitt (1987) and further described by Heckman & Vytlacil, 2007) and Heckman, Urzua, and Vytlacil (2006). In empirical work, MTEs have been used to estimate effects of breast cancer treatment (Basu, Heckman, Navarro‐Lozano, & Urzua, 2007), returns on education (Carneiro, Heckman, & Vytlacil, 2011), the effect of family size on quantity and quality of children (Brinch, Mogstad, & Wiswall, 2017), and the marginal returns of universal childcare (Cornelissen, Dustmann, Raute, & Schönberg, 2016). Péron and Dormont (2018) use MTEs to assess selection on moral hazard in supplementary health insurance. Considering that the decision to opt for a voluntary deductible might be related to the expected moral hazard response, we estimate MTEs to capture heterogeneity in response to the voluntary deductible and to test for the existence of selection on returns (e.g., on moral hazard).

Appendix S5 presents a detailed description of the estimation procedure for MTEs as an extension to the framework discussed previously. In our analysis, we use a parametric polynomial model to estimate MTEs (see Brave & Walstrum, 2014); we also present the results of the parametric normal and semiparametric models in Appendices S6–S10. The MTEs tell us how much higher or lower an individual's health care utilization is expected to be given a small increase in the propensity to not take out a voluntary deductible. In the polynomial model, Heckman et al. (2006) propose that the joint significance of the polynomial coefficients *ϕ*_*j*_ in equation (22) (see Appendix S5) reveals the presence of essential heterogeneity. In the case of the third‐degree polynomial, *ϕ*_1_ = *ϕ*_2_ = *ϕ*_3_ = 0 would mean that the treatment effect does not vary with unobservable characteristics; that is, that there would be no evidence of essential heterogeneity. In addition, ***β***_**0**_ captures the effect of observed individual characteristics on health care utilization without the voluntary deductible, whereas ***β***_**1**_ shows their effect with the voluntary deductible. A significant difference between ***β***_**1**_ and ***β***_**0**_ indicates a change in the impact of observed characteristics on health care utilization due to a higher deductible. In this model, the heterogeneity in moral hazard that stems from changes in the effects of regressors comes in addition to the heterogeneity that stems from unobserved characteristics.

## 6 RESULTS

### 6.1 Descriptive statistics

First, we investigate the descriptive statistics in Table 2, which shows means and standard deviations of all variables for the whole sample, for the subsample that has opted for the voluntary deductible, and for those who have no voluntary deductible. The last column provides the *p* value of a *t* test comparing the means of the two subsamples. We start by looking at the four health care utilization measures included in this analysis. As expected, we observe that individuals who select a voluntary deductible have, on average, fewer visits to a specialist, their GP, and mental health care providers and fewer days spent in the hospital than those who do not have a voluntary deductible. For visits to the specialist, GP, and days spent in the hospital, the difference is statistically significant at a 5% level. For visits to mental health care providers, the difference is not significant, but it has the same sign as other health care utilization variables. The raw data show that less insurance coverage is associated with significantly lower health care utilization. We consider this the first sign of asymmetric information for visits to the specialist and the GP and days spent in the hospital. For visits to mental health care providers, there is no evidence of asymmetric information.

Next, we compare the characteristics of individuals with and without a voluntary deductible. People who opt for a voluntary deductible are less risk averse, are more often male, are younger, are more highly educated, are more likely to be employed, are less likely to be married, have higher incomes, have better self‐assessed health, are less likely to have chronic diseases, and are more likely to smoke. These differences are statistically significant, suggesting that individuals who opt for a deductible are different from those who do not.

### 6.2 Multidimensional asymmetric information

Because Dutch insurers are not allowed to risk‐rate premiums, any individual characteristics that are correlated with both insurance coverage and health care utilization can be considered an indication of asymmetric information (Finkelstein & McGarry, 2006). We model self‐selection into a voluntary deductible and health care utilization more formally in Table 4.

**TABLE 4:** Ordinary least squares (OLS) estimates to test for selection and multidimensional asymmetric information

Variable	(1)	(2)	(3)	(4)	(5)
	Voluntary deductible	Health care utilization			
		Specialist visits	GP visits	Mental health care visits	Days in the hospital
Risk aversion	−0.0098^***^ (0.0032)	−0.0074 (0.0300)	−0.0159 (0.0302)	0.0335 (0.0270)	−0.0022 (0.0220)
Male	0.0253^**^ (0.0100)	−0.3039^***^ (0.0876)	−0.7317^***^ (0.1176)	−0.0684 (0.0754)	0.0111 (0.0675)
Age	0.0028 (0.0018)	−0.0473^**^ (0.0210)	−0.0301 (0.0224)	0.0412^*^ (0.0211)	−0.0137 (0.0172)
Age^2^	−0.00005^***^ (0.0000)	0.0007^***^ (0.0002)	0.0006^**^ (0.0002)	−0.0006^**^ (0.0002)	0.0002 (0.0002)
Employed	0.0162 (0.0116)	−0.2083^**^ (0.1053)	−0.2521^*^ (0.1300)	−0.3178^**^ (0.1553)	−0.2554^**^ (0.1300)
Educ.mid	−0.0036 (0.0118)	−0.2363^*^ (0.1259)	−0.2758^**^ (0.1250)	0.0633 (0.1249)	−0.0958 (0.0845)
Educ.high	0.0489^***^ (0.0134)	−0.1696 (0.1114)	−0.2332 (0.1420)	0.2320 (0.1478)	−0.0155 (0.1082)
Married	−0.0363^***^ (0.0140)	−0.0268 (0.1629)	−0.0029 (0.1304)	−0.0889 (0.0898)	−0.1266 (0.1089)
Good health	0.0598^***^ (0.0131)	−0.3616^***^ (0.0615)	−0.4396^***^ (0.0700)	−0.0579 (0.0599)	−0.1255^***^ (0.0466)
MHI‐5	−0.0006^*^ (0.0003)	−0.0190^***^ (0.0051)	−0.0339^***^ (0.0056)	−0.0425^***^ (0.0074)	−0.0129^**^ (0.0060)
Chronic cond.	−0.0507^***^ (0.0101)	1.2380^***^ (0.1269)	1.1802^***^ (0.1226)	0.1978^*^ (0.1026)	0.6009^***^ (0.1011)
Smokes	0.0132 (0.0130)	−0.0231 (0.1126)	−0.1939 (0.1318)	0.0535 (0.1064)	−0.0793 (0.0763)
Log‐income	0.0160 (0.0127)	−0.0640 (0.1316)	−0.3427^***^ (0.1103)	−0.1665 (0.1070)	0.0547 (0.0906)
Constant	0.0602 (0.1077)	4.0785^***^ (1.3272)	8.0109^***^ (1.1307)	4.6716^***^ (0.8390)	1.6114^**^ (0.7839)
*Average marginal effect of age*	−0.0023^***^ (0.0004)	0.0225^***^ (0.0049)	0.0320^***^ (0.0048)	−0.0215^***^ (0.0045)	0.0023 (0.0039)
*Observations*	14,089	14,089	14,089	14,089	14,089
*R‐square*	0.044	0.0409	0.0608	0.0346	0.0109

*Note*: Standard errors are clustered at the household level and presented in parentheses. The specifications include year dummies.

Abbreviations: GP, general practitioner; MHI, Mental Health Inventory.

^***^ *p* < 0.01.

^**^ *p* < 0.05.

^*^ *p* < 0.1.

Column 1 of Table 4 presents the coefficients of the pooled OLS, regressing the voluntary deductible on individual characteristics as in Equation 1. As we expected, the results show that more risk‐averse individuals are significantly less likely to select a voluntary deductible. In addition, older individuals, married people, and those with chronic conditions are less likely to opt for a voluntary deductible. Conversely, the probability of having a voluntary deductible is higher for males, highly educated people, and healthy individuals. This is in line with the results of van Winssen et al. (2015), who also find that these are the population groups who can benefit the most from opting for a voluntary deductible.

To investigate if any individual characteristics are correlated with both insurance coverage and health care utilization, we analyze Columns 2–5 of Table 4, which show the results from pooled OLS regressions with the four health care utilization measures as outcomes. Taken together with Column 1, these results show that being male and having good self‐assessed health is associated with a higher probability of having a voluntary deductible and lower utilization of specialists, a GP, and (only good health) hospital care.

The opposite is true for having a chronic condition; specifically, we observe a lower probability of having a voluntary deductible and higher utilization of all types of care. This suggests adverse selection and/or moral hazard in the utilization of specialists, a GP, and hospital care. The variable indicating the mental health status of the individual (MHI‐5) shows that better mental health is associated with a lower probability of having a voluntary deductible and also lower utilization of all types of care, including mental health care. This suggests advantageous selection on mental health care; in other words, healthier individuals select more insurance coverage even after controlling for risk aversion. Notably, the average marginal effects of age show adverse selection with regard to specialist and GP visits but, again, advantageous selection with regard to mental health care visits.

In summary, the test proposed by Finkelstein and McGarry (2006) indicates the presence of multidimensional private information. In the case of mental health care utilization, some dimensions lead to adverse selection and some lead to advantageous selection. As a result, the raw difference in mental health care utilization between individuals with and without a deductible is not significant (see Table 2).

### 6.3 Moral hazard in health care utilization

Having established asymmetric information and selection, we turn to estimating moral hazard in health care utilization. Using the models described in Equations 8–12, we model the decision to have any physician visits/hospital stays and the number of visits/days separately. ^4^For comparison, Appendix S2 presents the results of simple probit and OLS regressions, whereas Appendices S3 and S4 present the results from the same bivariate models as the main results but without an instrument because bivariate models are identified by their functional form. Controlling for sources of asymmetric information, such as risk aversion and risk type, a significant, negative relationship between the dummy for a voluntary deductible and the utilization variables can be considered evidence of moral hazard.

Table 5 presents the bivariate probit results, including an exclusion restriction. The first‐stage results indicate that supplementary insurance is a relevant instrument for the voluntary deductible because the coefficients are very significant. In the second stage results, the outcome is the probability of having any physician visits/days in the hospital.

**TABLE 5:** Voluntary deductible and the probability of having any physician visits: bivariate probit results

Variable	(1)	(2)	(3)	(4)
	Specialist visits	GP visits	Mental health care visits	Days in the hospital
First stage (voluntary deductible)				
Sup. insurance	−0.4493^***^ (0.0438)	−0.4361^***^ (0.0454)	−0.4273^***^ (0.0462)	−0.4253^***^ (0.0469)
Risk aversion	−0.0430^***^ (0.0126)	−0.0430^***^ (0.0128)	−0.0423^***^ (0.0127)	−0.0402^***^ (0.0127)
Control variables	Yes	Yes	Yes	Yes
Constant	Yes	Yes	Yes	Yes
Second stage (outcome (*P*(*Y* > 0)))				
Vol.deduct.	−1.0292^***^ (0.1271)	−0.6844^***^ (0.1706)	−0.6416^**^ (0.2550)	−1.228^***^ (0.1448)
Risk aversion	−0.0041 (0.0100)	0.0023 (0.0104)	−0.0067 (0.0172)	−0.0096 (0.0108)
Male	−0.1649^***^ (0.0345)	−0.4524^***^ (0.0350)	−0.1206^**^ (0.0549)	0.0273 (0.0349)
Age	−0.0145^**^ (0.0061)	−0.0106 (0.0066)	0.0060 (0.0109)	−0.0157^**^ (0.0065)
Age^2^	0.0003^***^ (0.0001)	0.0002^***^ (0.0001)	−0.0002^**^ (0.0001)	0.0002^**^ (0.0001)
Employed	−0.0638 (0.0403)	−0.0577 (0.0419)	−0.0179 (0.0633)	0.0235 (0.0426)
Educ.mid	0.0043 (0.0404)	0.0253 (0.0435)	0.1147^*^ (0.0697)	−0.0545 (0.0425)
Educ.high	0.1010^**^ (0.0439)	0.0892^*^ (0.0473)	0.2243^***^ (0.0717)	0.0536 (0.0441)
Married	−0.0911^**^ (0.0450)	0.0359 (0.0450)	−0.0643 (0.0636)	−0.0356 (0.0488)
Good health	−0.1807^***^ (0.0391)	−0.2080^***^ (0.0373)	−0.0476 (0.0693)	−0.0792 (0.0495)
MHI‐5	−0.0041^***^ (0.0011)	−0.0084^***^ (0.0012)	−0.0259^***^ (0.0017)	−0.004^***^ (0.0012)
Chronic cond.	0.5628^***^ (0.0397)	0.3761^***^ (0.0395)	0.1597^**^ (0.0648)	0.2273^***^ (0.0477)
Smokes	−0.0829^**^ (0.0403)	−0.1154^***^ (0.0413)	0.0437 (0.0638)	−0.0143 (0.0425)
Log‐income	0.0553 (0.0379)	−0.0465 (0.0396)	−0.0884 (0.0603)	0.0297 (0.0407)
Constant	−0.2373 (0.3063)	1.7446^***^ (0.3320)	1.4112^***^ (0.4853)	−0.6134^*^ (0.3362)
*Observations*	14,089	14,089	14,089	14,089
*ρ (rho)*	0.506^***^	0.307^***^	0.389^**^	0.689^***^
*Log‐likelihood*	−14,407	−13,775	−8,769	−10,611
*Average marginal effect vol.deduct.*	−0.3143^***^	−0.2337^***^	−0.0552^**^	−0.163^***^

*Note*: Standard errors are clustered at the household level and presented in parentheses. Both stages of the specifications include wave dummies.

Abbreviations: GP, general practitioner; MHI, Mental Health Inventory.

^***^ *p* < 0.01.

^**^ *p* < 0.05.

^*^ *p* < 0.1.

The parameter *ρ* at the bottom of Table 5 measures the correlation of the residuals of the first‐ and second‐stage models. For all four models, this correlation is highly significant, indicating that endogeneity is an important concern.

Turning to the coefficients, we observe a significant, negative effect of having a voluntary deductible on the probability of visiting a specialist, a GP, or a mental health care specialist or being hospitalized. We present the average marginal effects at the bottom of Table 5 and show that the effects of the voluntary deductible are large. Having a voluntary deductible decreases the probability of any specialist visits by 31 percentage points (p.p.), GP visits by 23 p.p., mental health care specialist visits by 5.5 p.p., and hospitalizations by 16 p.p.

Table 6 presents the results of the log‐linear regression with the endogenous treatment variable, including an exclusion restriction. Again, first‐stage results indicate that supplementary insurance is a relevant instrument for having the voluntary deductible. In the second stage results, the outcome is the logarithm of the number of physician visits/days in the hospital. The parameter rho shows that the residuals of the first‐ and second‐stage equations are correlated for the number of specialist visits, GP visits, and days in hospital, indicating endogeneity. The residuals are not correlated for mental health care visits.

**TABLE 6:** Voluntary deductible and the number of physician visits (if positive): log‐linear regression with endogenous treatment (estimated by quasi‐maximum likelihood)

Variable	(1)	(2)	(3)	(4)
	Specialist visits	GP visits	Mental health care visits	Days in the hospital
First stage (voluntary deductible)				
Sup. insurance	−0.3445^***^ (0.0670)	−0.3898^***^ (0.0513)	−0.6990^***^ (0.1382)	−0.2140^**^ (0.0865)
Risk aversion	−0.0343^**^ (0.0174)	−0.0434^***^ (0.0140)	−0.0622^*^ (0.0368)	−0.0786^***^ (0.0266)
Control variables	Yes	Yes	Yes	Yes
Constant	Yes	Yes	Yes	Yes
Second stage (outcome (*ln* (number of visits/days))				
Vol.deduct.	−0.1870^**^ (0.0854)	−0.2714^***^ (0.0602)	−0.3524 (0.3123)	1.5449^***^ (0.1116)
Risk aversion	−0.0053 (0.0079)	0.0021 (0.0064)	0.0491^**^ (0.0230)	0.0064 (0.0194)
Male	−0.0426 (0.0276)	−0.1496^***^ (0.0217)	−0.0003 (0.0818)	0.0207 (0.0658)
Age	−0.0102^**^ (0.0050)	−0.0047 (0.0039)	0.0254^*^ (0.0135)	−0.0085 (0.0112)
Age^2^	0.0001^**^ (0.0000)	0.0001^***^ (0.0000)	−0.0003^**^ (0.0001)	0.0002 (0.0001)
Employed	−0.0653^**^ (0.0326)	−0.0595^**^ (0.0265)	−0.0257 (0.1047)	−0.2597^***^ (0.0821)
Educ.mid	−0.0805^**^ (0.0360)	−0.0454^*^ (0.0275)	−0.0494 (0.1089)	−0.1341^*^ (0.0771)
Educ.high	−0.0694^**^ (0.0353)	−0.0693^**^ (0.0288)	0.1643 (0.1224)	−0.2028^**^ (0.0814)
Married	0.0130 (0.0356)	−0.0128 (0.0294)	0.0346 (0.1109)	−0.1337 (0.0862)
Good health	−0.1536^***^ (0.0302)	−0.1511^***^ (0.0226)	−0.1246 (0.0978)	−0.0549 (0.0870)
MHI‐5	−0.0052^***^ (0.0009)	−0.0069^***^ (0.0008)	−0.0088^***^ (0.0023)	−0.0024 (0.0023)
Chronic cond.	0.2566^***^ (0.0267)	0.2159^***^ (0.0232)	−0.0554 (0.0824)	0.2354^***^ (0.0634)
Smokes	0.0137 (0.0345)	−0.0563^**^ (0.0281)	0.0702 (0.0959)	0.0259 (0.0814)
Log‐income	−0.0611^**^ (0.0294)	−0.0706^***^ (0.0267)	−0.1201 (0.0989)	0.0121 (0.0857)
Constant	1.9740^***^ (0.2622)	2.0009^***^ (0.2215)	2.4867^***^ (0.7831)	0.9775 (0.7514)
*Observations*	5,712	9,783	856	1,506
*rho*	0.142^***^	0.205^***^	0.101	−0.850^***^
*sigma*	0.750	0.693	0.956	1.043
*lambda*	0.106	0.142	0.0968	−0.886
*SE lambda*	0.0360	0.0282	0.177	0.0627
*Log‐likelihood*	−8,487	−14,060	−1,539	−2,476

*Note*: Standard errors are clustered at the household level and presented in parentheses. Both stages of the specifications include wave dummies.

^***^ *p* < 0.01.

^**^ *p* < 0.05.

^*^ *p* < 0.1.

Next, we consider the effect of having a voluntary deductible. The coefficients show that having a voluntary deductible reduces the number of specialist visits by approximately 18.7% and the number of GP visits by approximately 27.1%, ceteris paribus. The significant effect on GP visits seems surprising at first because GP visits are excluded from the deductible. However, because the GP acts as a gatekeeper for other kinds of care that are subject to the deductible, it is possible that having the deductible reduces visits to the GP that would be made with the intention of getting a referral to a specialist. The effect on the number of mental health care visits is not statistically significant. Notably, the coefficient of the voluntary deductible is positive in the model for the number of days spent in the hospital (Column 4). This means that conditional on having any hospitalization, people with a deductible spend 155% longer in the hospital. Because they are less likely to be hospitalized, these individuals seem to avoid hospitalization for less severe issues and go to the hospital only for more serious health problems. Interestingly, risk aversion does not seem to be significantly associated with health care utilization, except for the number of mental health care visits, even though it is a significant determinant of selection into the voluntary deductible (first stage). As we expected, health care utilization seems to be driven mostly by individuals' health status. Altogether, it seems that voluntary deductibles help reduce moral hazard, especially on the extensive margin.

### 6.4 Selection on moral hazard

The models estimated in the previous section assume that the moral hazard effect is homogeneous across individuals. In this section, we test this assumption by allowing for both heterogeneities in the effects of regressors and heterogeneity that stems from the unobserved propensity to opt for a voluntary deductible. For this purpose, we estimate the MTEs of having a voluntary deductible on the natural logarithm of our four health care utilization measures on the sample of individuals who have a positive number of visits/hospital days using a parametric polynomial specification. We compute standard errors by bootstrapping and cluster at the household level.

**FIGURE 1:**
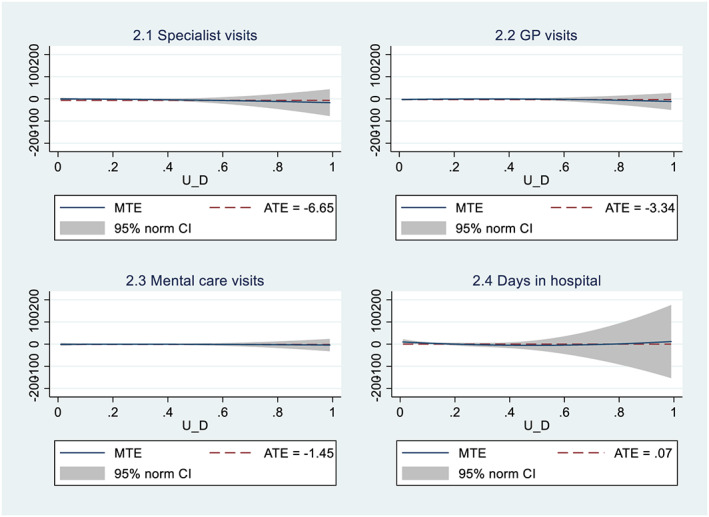
MTEs of polynomial (3) model. ATE, average treatment effect; GP, general practitioner; MTE, marginal treatment effect [Colour figure can be viewed at wileyonlinelibrary.com]

The results presented in Table 7 show no evidence of selection on moral hazard, because the polynomial terms p1, p2, and p3 are not individually or jointly significant for any of the outcome variables. The average treatment effect of the voluntary deductible (*E*(*Y*_1_–*Y*_0_)) appears to be imprecisely estimated but has the same signs as in Table 6. In addition, Figure 1 shows that the MTEs do not differ from zero at any point of the distribution of the propensity not to take out a voluntary deductible (*u*_*d*_). The results from the parametric normal and semiparametric models in Appendices S5–S10 also show no evidence of selection on moral hazard, but they estimate the treatment effects more precisely. We conclude that selection on moral hazard is not an issue in the choice of a voluntary deductible in the Dutch health insurance system. Individuals do not seem to choose the size of their deductible based on potential changes in their health care consumption due to a larger deductible.

**TABLE 7:** Effect of covariates on health care utilization and moral hazard of having a voluntary deductible: parametric polynomial specification

Variable	(1)	(2)	(3)	(4)	(5)	(6)	(7)	(8)
	Specialist visits		GP visits		Mental health care visits		Days in the hospital	
	*β*_0_	(*β*_1_–*β*_0_)	*β*_0_	(*β*_1_–*β*_0_)	*β*_0_	(*β*_1_–*β*_0_)	*β*_0_	(*β*_1_–*β*_0_)
Risk aversion	−0.009 (0.023)	−0.028 (0.144)	−0.012 (0.017)	0.039 (0.083)	0.090 (0.057)	−0.221 (0.218)	0.099^*^ (0.059)	−0.519 (0.444)
Male	0.003 (0.053)	−0.383 (0.349)	−0.159^***^ (0.049)	0.155 (0.251)	0.147 (0.132)	−0.973^*^ (0.549)	0.218^*^ (0.129)	−1.404 (0.870)
Age	−0.006 (0.011)	−0.018 (0.086)	0.000 (0.008)	−0.008 (0.044)	0.002 (0.020)	0.144 (0.115)	−0.030 (0.020)	0.239 (0.167)
Age2	0.000 (0.000)	0.000 (0.001)	0.000 (0.000)	0.000 (0.000)	−0.000 (0.000)	−0.001 (0.001)	0.000^**^ (0.000)	−0.003 (0.002)
Employed	−0.037 (0.098)	0.033 (0.578)	−0.002 (0.069)	−0.271 (0.404)	0.273 (0.189)	−1.657^*^ (0.856)	−0.528^**^ (0.227)	1.103 (1.928)
Educ.mid	−0.002 (0.091)	−0.839 (0.637)	−0.009 (0.049)	−0.289 (0.353)	−0.345^**^ (0.153)	1.506^**^ (0.737)	−0.082 (0.136)	−0.542 (1.089)
Educ.high	−0.020 (0.107)	−0.244 (0.721)	0.054 (0.086)	−0.532 (0.445)	−0.043 (0.179)	1.021 (0.850)	−0.481^***^ (0.173)	1.454 (1.173)
Married	0.037 (0.080)	−0.356 (0.498)	−0.085 (0.056)	0.373 (0.302)	−0.264 (0.199)	1.603^*^ (0.862)	−0.234 (0.170)	0.701 (1.068)
Good health	−0.188^**^ (0.079)	0.330 (0.436)	−0.174^***^ (0.065)	0.267 (0.273)	−0.727^***^ (0.209)	2.707^***^ (0.864)	−0.115 (0.171)	0.279 (0.986)
MHI5	−0.009^***^ (0.002)	0.030^*^ (0.016)	−0.005^***^ (0.001)	−0.014^*^ (0.008)	−0.017^***^ (0.004)	0.046^**^ (0.020)	−0.002 (0.005)	−0.003 (0.031)
Chronic cond.	0.292^***^ (0.081)	−0.633 (0.642)	0.240^***^ (0.071)	−0.581 (0.401)	−0.152 (0.174)	0.493 (0.861)	0.424^***^ (0.165)	−1.028 (1.322)
Smokes	−0.149^*^ (0.080)	1.258^**^ (0.498)	−0.092 (0.062)	0.328 (0.320)	−0.006 (0.196)	0.511 (0.736)	0.176 (0.110)	−1.121 (0.793)
Log income	−0.108 (0.069)	0.280 (0.409)	−0.108^**^ (0.049)	0.215 (0.243)	−0.084 (0.201)	−0.267 (0.821)	0.200 (0.188)	−0.970 (1.323)
p1	−3.270 (5.505)		−3.672 (3.302)		−4.966 (8.993)		15.872 (16.275)	
p2	−1.659 (14.487)		8.507 (10.019)		1.911 (11.305)		−32.470 (37.379)	
p3	−4.607 (18.431)		−8.703 (12.295)		−2.450 (10.606)		22.289 (49.657)	
*Joint test p1 p2 p3, p value*		0.4001		0.7363		0.8568		0.4712
*Joint test (β*_*1−*_ *β*_*0*_ *), p value*		0.1116		0.1975		0.0000		0.6611
*E(Y1‐Y0)@X*		−6.649 (8.063)		−3.343 (4.855)		−1.445 (3.409)		0.075 (21.823)
Constant	2.532^***^ (0.714)		2.353^***^ (0.473)		3.340^**^ (1.703)		−1.136 (2.040)	
*Observations*	5,712	5,712	9,783	9,783	856	856	1,506	1,506

*Note*: The generalized Roy model is fit in two stages: a first‐stage probit regression to obtain the propensity score *p*, followed by a linear regression of log‐health care utilization measure on a constant, covariates, and their interactions with *p,* and the polynomial terms of *p* (p1, p2, and p3). Bootstrapped standard errors clustered at the household level are presented in parentheses. The specifications include wave dummies.

^***^ *p* < 0.01.

^**^ *p* < 0.05.

^*^ *p* < 0.1.

In addition, for the three types of physical health care (specialist visits, GP visits, and hospitalization days), the (*β*_1_–*β*_0_) terms are jointly insignificant, indicating homogeneity in the effects of the explanatory variables between those with and without a voluntary deductible. This suggests that the results from Section 6.3 are robust. Nevertheless, for the utilization of mental health care, the terms (*β*_1_–*β*_0_) are jointly significant, suggesting that the utilization of mental health care for people with a voluntary deductible is different relative to the same characteristics than it is for the people without a deductible.

## 7 CONCLUSIONS

This paper uses the LISS panel data from the Netherlands to investigate whether voluntary deductibles in the Dutch health insurance system reduce moral hazard or act as a premium reduction tool for low‐risk individuals. The descriptive statistics show that less insurance coverage is indeed associated with significantly lower health care utilization, except for mental health care. The test for multidimensional asymmetric information by Finkelstein and McGarry (2006) indicates that although the three types of physical health care utilization exhibit adverse selection, mental health care utilization is subject to advantageous selection.

The results of our analysis of selection into a deductible show that risk‐averse individuals are significantly less likely to opt for a voluntary deductible. Moreover, older individuals, married people, and those with chronic conditions are less likely to opt for a voluntary deductible, whereas the probability of having a voluntary deductible is higher for males, highly educated people, and healthy people. These findings are in line with the results of van Winssen et al. (2015), who show that these population groups benefit the most from a voluntary deductible.

Furthermore, we show that a voluntary deductible is an effective tool for reducing moral hazard in the Dutch health insurance system. Moreover, our results indicate that it is important to model both the decision to use any health care and the decision about the amount of health care to use, as the effects differ between the two decisions. Although having a voluntary deductible reduces the probability of using health care, the effects on the amount of utilization differ. We do not find evidence of selection on moral hazard; that is, individuals do not seem to consider their potential change in behavior when choosing a deductible.

Remarkably, the effect sizes are large, which is common in the literature (e.g., Gardiol et al., 2005). Having a voluntary deductible decreases the probability of specialist visits by 31 p.p., GP visits by 23 p.p., mental health care specialist visits by 5.5 p.p., and hospitalizations by 16 p.p. Considering the amount of health care utilization, having a voluntary deductible reduces the number of specialist visits by approximately 19.7% and the number of GP visits by approximately 27.1%. It seems that mental health care utilization responds to incentives in a different way than physical health care because the effect on the number of mental health care visits is not statistically significant. Notably, a voluntary deductible has a positive effect on the number of days spent in the hospital, conditional on having any hospitalization. Individuals with a deductible spend 155% longer time in the hospital than those without. It seems that individuals who opt for a voluntary deductible avoid hospitalizations for less severe issues and go to the hospital only for more serious health problems. Another possibility is that they postpone hospitalization until the moment that the health problem becomes more severe.

In summary, even though a voluntary deductible creates incentives for adverse (or in the case of mental health care, advantageous) selection, it is an effective tool for reducing moral hazard in health care utilization. These effects seem universal on the extensive margin (having any physician visits/hospitalizations) but differ between the types of health care on the intensive margin (amount of utilization).

## Supporting information


**Data S1.** Supporting Information

**Data S2.** Supporting Information
