# Supplementary material for: Moral hazard and selection for voluntary deductibles
Source: Health Econ. 2020 Jul 31;29(10):1251–69. doi: 10.1002/hec.4134 (PMC7539990; doi:10.1002/hec.4134)
Supplement: Supplementary file 2 — Data S2. Supporting Information [file HEC-29-1251-s002.zip › Appendices_S1-S6&S8.docx]

Appendix S1: List of variables

| Variable name | Description |
| --- | --- |
| *Health care utilization (outcomes)* |  |
| Specialist visits | Number of specialist visits in last 12 months |
| GP visits | Number of GP visits in past 12 months |
| Mental health care visits | Number of mental health care visits in the past 12 months |
| Days in the hospital | Days spent in the hospital when last hospitalized in last 12 months |
| *Deductible* |  |
| Vol.deduct | Has a voluntary deductible |
| Vol.deduct size | Size of the voluntary deductible |
| Man.deduct | Size of the mandatory deductible |
| Total deduct | Total deductible size |
| *Sociodemographic variables from November* *one year earlier (before the insurance for the following year is chosen)* | |
| Risk aversion | Risk aversion (1–5) calculated as number of safe choices in five lottery games |
| Male | Male |
| Age | Age |
| Educ.low | Up to intermediate secondary education |
| Educ.mid | Secondary or vocational education |
| Educ.high | College or university |
| Employed | Employed |
| Married | Married |
| Log-income | Log of household income |
| *Lagged health variables:* |  |
| Good health | Very good or excellent self-assessed health |
| MHI-5 | Mental health inventory on 0–100 scale (100 = perfect health) |
| Chronic cond. | Has a chronic condition |
| Smokes | Currently smokes |

Appendix S2: Voluntary deductible and physician visits: OLS and probit results

|  | (1) | (2) | (3) | (4) | (6) | (7) | (8) | (9) |
| --- | --- | --- | --- | --- | --- | --- | --- | --- |
| Variables | Specialist visits | GP visits | Mental health care visits | Days in the hospital | Specialist visits | GP visits | Mental health care visits | Days in the hospital |
|  | *Probit (outcome (P(Y* *>* *0**)))* | | | | *OLS (outcome (ln (number of visits/days**))* | | | |
| Vol.deduct. | −0.1693*** (0.0382) | −0.1494*** (0.0369) | 0.0024 (0.0599) | −0.1292*** (0.0496) | 0.0096 (0.0347) | −0.0155 (0.0239) | −0.1885** (0.0950) | −0.0783 (0.0709) |
| Risk aversion | 0.0051 (0.0099) | 0.0077 (0.0103) | 0.0011 (0.0170) | 0.0059 (0.0109) | −0.0039 (0.0079) | 0.0045 (0.0064) | 0.0512** (0.0225) | −0.0238 (0.0159) |
| Male | −0.1988*** (0.0349) | −0.4757*** (0.0343) | −0.1444*** (0.0545) | −0.0074 (0.0366) | −0.0422 (0.0276) | −0.1543*** (0.0216) | 0.0037 (0.0813) | −0.0065 (0.0556) |
| Age | −0.0173*** (0.0063) | −0.0122* (0.0067) | 0.0033 (0.0110) | −0.0226*** (0.0065) | −0.0104** (0.0050) | −0.0049 (0.0039) | 0.0246* (0.0135) | −0.0070 (0.0107) |
| Age^2^ | 0.0003*** (0.0001) | 0.0003*** (0.0001) | −0.0002 (0.0001) | 0.0003*** (0.0001) | 0.0001** (0.0000) | 0.0001*** (0.0000) | −0.0003** (0.0001) | 0.0001 (0.0001) |
| Employed | −0.0846** (0.0407) | −0.0673 (0.0423) | −0.0300 (0.0647) | −0.0055 (0.0443) | −0.0740** (0.0327) | −0.0644** (0.0262) | −0.0332 (0.1040) | −0.1644** (0.0714) |
| Educ.mid | 0.0074 (0.0417) | 0.0278 (0.0446) | 0.1209* (0.0707) | −0.0585 (0.0450) | −0.0774** (0.0360) | −0.0445 (0.0274) | −0.0457 (0.1093) | −0.1411** (0.0682) |
| Educ.high | 0.0611 (0.0448) | 0.0637 (0.0472) | 0.1956*** (0.0742) | −0.0169 (0.0451) | −0.0751** (0.0353) | −0.0827*** (0.0285) | 0.1634 (0.1237) | −0.1383* (0.0713) |
| Married | −0.0622 (0.0456) | 0.0571 (0.0448) | −0.0388 (0.0634) | 0.0145 (0.0499) | 0.0174 (0.0355) | −0.0080 (0.0293) | 0.0332 (0.1114) | −0.1275* (0.0730) |
| Good health | −0.2508*** (0.0376) | −0.2450*** (0.0356) | −0.1002 (0.0651) | −0.1900*** (0.0461) | −0.1574*** (0.0303) | −0.1610*** (0.0224) | −0.1394 (0.0957) | −0.0125 (0.0676) |
| MHI-5 | −0.0038*** (0.0012) | −0.0083*** (0.0012) | −0.0264*** (0.0016) | −0.0035*** (0.0013) | −0.0051*** (0.0010) | −0.0067*** (0.0007) | −0.0088*** (0.0023) | −0.0030 (0.0021) |
| Chronic cond. | 0.6417*** (0.0372) | 0.4101*** (0.0387) | 0.2089*** (0.0566) | 0.3508*** (0.0376) | 0.2657*** (0.0269) | 0.2278*** (0.0229) | −0.0511 (0.0817) | 0.1810*** (0.0550) |
| Smokes | −0.0995** (0.0404) | −0.1249*** (0.0410) | 0.0356 (0.0649) | −0.0392 (0.0453) | 0.0114 (0.0347) | −0.0619** (0.0280) | 0.0587 (0.0955) | 0.0025 (0.0689) |
| Log-income | 0.0437 (0.0386) | −0.0564 (0.0399) | −0.1046* (0.0607) | 0.0106 (0.0410) | −0.0582** (0.0295) | −0.0692*** (0.0267) | −0.1018 (0.0946) | −0.0511 (0.0744) |
| Constant | −0.3193 (0.3100) | 1.7457*** (0.3354) | 1.4595*** (0.4891) | −0.7448** (0.3396) | 1.9152*** (0.2639) | 1.9413*** (0.2219) | 2.3309*** (0.7320) | 1.7944*** (0.6539) |
| *Observations* | *14,089* | *14,089* | *14,089* | *14,089* | *5,712* | *9,783* | *856* | *1,506* |
| *Log-likelihood* | *−8,413* | *−7,769* | *−2,761* | *−4,615* |  |  |  |  |
| *R-square* |  |  |  |  | *0.0764* | *0.1352* | *0.0760* | *0.0939* |

Notes: Standard errors are clustered at the household level and presented in parentheses. All specifications include wave dummies. ****p* < 0.01, ***p* < 0.05, *p < 0.1.

Appendix S3: Voluntary deductible and the probability of having any physician visits: bivariate probit results without an instrument

|  | (1) | (2) | (3) | (4) |
| --- | --- | --- | --- | --- |
| Variables | Specialist visits | GP visits | Mental health care visits | Days in the hospital |
| *First stage (voluntary deductible)* | | | | |
| Risk aversion | −0.0411*** (0.0128) | −0.0403*** (0.0134) | −0.0403*** (0.0128) | −0.0381*** (0.0127) |
| Control variables | Yes | Yes | Yes | Yes |
| Constant | Yes | Yes | Yes | Yes |
| *Second stage (outcome (P(Y* *>* *0**)))* | | | | |
| Vol.deduct. | −0.5846* (0.3453) | 0.0559 (1.5110) | −0.7964*** (0.2087) | −1.4599*** (0.1235) |
| Risk aversion | 0.0010 (0.0107) | 0.0098 (0.0191) | −0.0092 (0.0169) | −0.0156 (0.0106) |
| Male | −0.1858*** (0.0371) | −0.4797*** (0.0392) | −0.1115** (0.0542) | 0.0420 (0.0340) |
| Age | −0.0160** (0.0063) | −0.0129 (0.0084) | 0.0070 (0.0106) | −0.0114* (0.0066) |
| Age^2^ | 0.0003*** (0.0001) | 0.0003*** (0.0001) | −0.0002** (0.0001) | 0.0001 (0.0001) |
| Employed | −0.0767* (0.0415) | −0.0705 (0.0467) | −0.0129 (0.0620) | 0.0337 (0.0413) |
| Educ.mid | 0.0060 (0.0413) | 0.0284 (0.0451) | 0.1100 (0.0684) | −0.0493 (0.0415) |
| Educ.high | 0.0808* (0.0479) | 0.0529 (0.0963) | 0.2308*** (0.0711) | 0.0806* (0.0439) |
| Married | −0.0766 (0.0472) | 0.0649 (0.0736) | −0.0727 (0.0616) | −0.0599 (0.0487) |
| Good health | −0.2224*** (0.0468) | −0.2566*** (0.0891) | −0.0305 (0.0650) | −0.0185 (0.0564) |
| MHI-5 | −0.0040*** (0.0012) | −0.0081*** (0.0018) | −0.0253*** (0.0017) | −0.0038*** (0.0012) |
| Chronic cond. | 0.6130*** (0.0468) | 0.4201*** (0.0773) | 0.1403** (0.0631) | 0.1541** (0.0614) |
| Smokes | −0.0928** (0.0412) | −0.1273*** (0.0439) | 0.0468 (0.0631) | 0.0002 (0.0414) |
| Log-income | 0.0497 (0.0388) | −0.0597 (0.0464) | −0.0809 (0.0577) | 0.0391 (0.0406) |
| athrho | 0.2396 (0.2105) | −0.1168 (0.8694) | 0.5239*** (0.1718) | 1.2896*** (0.3665) |
| Constant | −0.2875 (0.3099) | 1.7312*** (0.3614) | 1.3505*** (0.4672) | −0.6042* (0.3340) |
| *Observations* | *14,089* | *14,089* | *14,089* | *14,089* |
| *ρ (rho)* | *0.235****** | *−0.116****** | *0.481****** | *0.859****** |
| *Log-likelihood* | *−14,520* | *−13,876* | *−8,867* | *−10,718* |
| *Average marginal effect vol.deduct.* | *−0.1892** | *0.0173* | *−0.0679****** | *−0.2064****** |

Notes: Standard errors are clustered at the household level and presented in parentheses. All specifications include wave dummies. ****p* < 0.01, ***p* < 0.05, **p* < 0.1.

Appendix S4. Voluntary deductible and the number of physician visits (if positive): log-linear regression with endogenous treatment without an instrument

|  | (1) | (2) | (3) | (4) |
| --- | --- | --- | --- | --- |
| Variables | Specialist visits | GP visits | Mental health care visits | Days in the hospital |
| *First stage (voluntary deductible)* | | | | |
| Risk aversion | −0.0340* (0.0174) | −0.0416*** (0.0140) | −0.0560 (0.0378) | −0.0817*** (0.0264) |
| Control variables | Yes | Yes | Yes | Yes |
| Constant | Yes | Yes | Yes | Yes |
| *Second stage (outcome (ln (number of visits/days**))* | | | | |
| Vol.deduct. | −0.0720 (0.1215) | −0.1519* (0.0842) | −0.3016 (0.7843) | 1.5512*** (0.1141) |
| Risk aversion | −0.0045 (0.0079) | 0.0032 (0.0064) | 0.0497** (0.0243) | 0.0066 (0.0194) |
| Male | −0.0424 (0.0276) | −0.1518*** (0.0216) | 0.0010 (0.0837) | 0.0208 (0.0659) |
| Age | −0.0103** (0.0050) | −0.0048 (0.0039) | 0.0252* (0.0136) | −0.0085 (0.0113) |
| Age^2^ | 0.0001** (0.0000) | 0.0001*** (0.0000) | −0.0003** (0.0001) | 0.0002 (0.0001) |
| Employed | −0.0704** (0.0328) | −0.0618** (0.0263) | −0.0280 (0.1145) | −0.2601*** (0.0822) |
| Educ.mid | −0.0787** (0.0360) | −0.0449 (0.0274) | −0.0483 (0.1092) | −0.1341* (0.0771) |
| Educ.high | −0.0727** (0.0355) | −0.0756*** (0.0291) | 0.1640 (0.1223) | −0.2031** (0.0815) |
| Married | 0.0156 (0.0354) | −0.0105 (0.0293) | 0.0342 (0.1108) | −0.1337 (0.0862) |
| Good health | −0.1558*** (0.0302) | −0.1557*** (0.0226) | −0.1292 (0.1172) | −0.0550 (0.0871) |
| MHI-5 | −0.0051*** (0.0009) | −0.0068*** (0.0007) | −0.0088*** (0.0023) | −0.0024 (0.0023) |
| Chronic cond. | 0.2619*** (0.0271) | 0.2214*** (0.0231) | −0.0541 (0.0836) | 0.2356*** (0.0635) |
| Smokes | 0.0123 (0.0346) | −0.0589** (0.0280) | 0.0667 (0.1105) | 0.0260 (0.0814) |
| Log-income | −0.0594** (0.0294) | −0.0699*** (0.0267) | −0.1144 (0.1267) | 0.0124 (0.0857) |
| Constant | 1.9396*** (0.2630) | 1.9731*** (0.2213) | 2.4385** (1.0187) | 0.9744 (0.7520) |
| *Observations* | *5,712* | *9,783* | *856* | *1,506* |
| *Rho* | *0.0584* | *0.108** | *0.0673* | *−0.849****** |
| *sigma* | *0.748* | *0.689* | *0.955* | *1.044* |
| *lambda* | *0.0437* | *0.0747* | *0.0643* | *−0.886* |
| *SE lambda* | *0.0573* | *0.0419* | *0.426* | *0.0632* |
| *Log-likelihood* | *−8,505* | *−14,108* | *−1,553* | *−2,479* |

Notes: Models estimated by quasi-maximum likelihood. Standard errors are clustered at the household level and presented in parentheses. All specifications include wave dummies. ****p* < 0.01, ***p* < 0.05, **p* < 0.1.

Appendix S5: Estimation of marginal treatment effects (MTEs)

We start with the potential outcome framework presented in equations (3)–(7). Allowing for heterogeneity, $v_{it}$in equation (6) becomes an unobservable random variable corresponding to the individual idiosyncratic disutility of choosing a voluntary deductible (e.g., disutility of administrative switching costs). Notice that the binary decision model is equivalent to

$d_{it}=\left\{ \begin{aligned} 1 if \boldsymbol{x}_{\boldsymbol{it}}^{\mathbf{'}}\boldsymbol{\beta}_{\boldsymbol{d}}+ \gamma z_{it}> v_{it} \\ 0 if \boldsymbol{x}_{\boldsymbol{it}}^{\mathbf{'}}\boldsymbol{\beta}_{\boldsymbol{d}}+ \gamma z_{it}\leq v_{it} \end{aligned} \right.$, (13)

where transforming equation (13) by using the cumulative distribution of $v_{it}$, F$(v_{it})$, yields the following propensity score function, $P\left( z_{it}, \boldsymbol{x}_{\boldsymbol{it}} \right)$:

$F_{v}\left( \boldsymbol{x}_{\boldsymbol{it}}^{\mathbf{'}}\boldsymbol{\beta}_{\boldsymbol{d}}+ \gamma z_{it} \right)>F_{v}(v_{it})$ (14)

$P\left( z_{it}, \boldsymbol{x}_{\boldsymbol{it}} \right)\mathbf{>}u_{itd}$,

where $u_{itd}$ is a uniformly distributed random variable between 0 and 1 and serves as a standardized measure of a person's unobservable propensity not to take out a voluntary deductible. Individuals who have a $u_{itd}$ close to 1 exhibit a large unobservable propensity to avoid the voluntary deductible.

The MTE is defined as the marginal return of treatment ${(d}_{it}=1)$ conditional on $\boldsymbol{x}_{\boldsymbol{it}}$and the propensity not to be treated ($u_{itd}$), as shown in Björklund and Moffitt (1987):

$MTE \equiv E\left( y_{it1} - y_{it0} \right| \boldsymbol{x}_{\boldsymbol{it}},{z_{it},u}_{itd})$. (15)

By assuming that ($u_{it1}$,$u_{it0}$, $v_{it}$) ∼ N(0, *Σ*), where *Σ* is the variance–covariance matrix of the three unobservables, we can estimate the MTE over the range of *P*($z_{it}$), that is, (0, 1). The propensity score $P\left( z_{it} \right)$ is generated from a probit model. Then,

$MTE\left( \boldsymbol{x}_{\boldsymbol{it}},u_{itd} \right)= \boldsymbol{x}_{\boldsymbol{it}}^{\mathbf{'}}{\mathbf{(}\boldsymbol{\beta}}_{\mathbf{1}}-\boldsymbol{\beta}_{\mathbf{0}}) + (\rho_{1} - \rho_{0})\Phi^{-1}(u_{itd})$, (16)

such that *ρ_j_*, *j* = (0, 1), corresponds to the element of *Σ* containing the covariance between $u_{itd}$ and $v_{it}$, and *Φ* is the cumulative normal distribution. Estimation of the parameters of the MTE then follows from the conditional expectations of $y_{it}$ by treatment status:

$E\{y_{it}|\boldsymbol{x}_{\boldsymbol{it}}, P\left( z_{it}, \boldsymbol{x}_{\boldsymbol{it}} \right), d_{it}=1\} =\boldsymbol{x}_{\boldsymbol{it}}^{\mathbf{'}}\boldsymbol{\beta}_{\mathbf{1}} + E\{u_{it1}|\boldsymbol{x}_{\boldsymbol{it}}, P\left( z_{it}, \boldsymbol{x}_{\boldsymbol{it}} \right), d_{it}=1\}$, (17)

$E\{y_{it}|\boldsymbol{x}_{\boldsymbol{it}}, P\left( z_{it}, \boldsymbol{x}_{\boldsymbol{it}} \right), d_{it}=0\} =\boldsymbol{x}_{\boldsymbol{it}}^{\mathbf{'}}\boldsymbol{\beta}_{\mathbf{0}} + E\{u_{it0}|\boldsymbol{x}_{\boldsymbol{it}}, P\left( z_{it}, \boldsymbol{x}_{\boldsymbol{it}} \right), d_{it}=0\}$. (18)

This approach is referred to as “parametric normal” (Brave and Valstrum, 2014). Following Heckman and Vytlacil (2007), equations (17)–(18) can be written as follows:

$E\{y_{it}|\boldsymbol{x}_{\boldsymbol{it}}, P\left( z_{it}, \boldsymbol{x}_{\boldsymbol{it}} \right)=p\} = \boldsymbol{x}_{\boldsymbol{it}}^{'}\boldsymbol{\beta}_{\mathbf{0}} + \boldsymbol{x}_{\boldsymbol{it}}^{'}{(\boldsymbol{\beta}}_{\mathbf{1}}-\boldsymbol{\beta}_{\mathbf{0}})p + K(p)$, (19)

$K(p) = E\{u_{it0}|P\left( z_{it}, \boldsymbol{x}_{\boldsymbol{it}} \right)\mathbf{=}p\} + E\{u_{it1}-u_{it0}|P\left( z_{it}, \boldsymbol{x}_{\boldsymbol{it}} \right)\}p$.

If we partially relax the assumption of joint normality, we can arrive at the polynomial parametric model. Given an estimate of $P\left( z_{it}, \boldsymbol{x}_{\boldsymbol{it}} \right)$ from equation (14), equation (21) can be rewritten as follows:

$E\{y_{it}|\boldsymbol{x}_{\boldsymbol{it}}, P\left( z_{it}, \boldsymbol{x}_{\boldsymbol{it}} \right)\mathbf{=}p\} = \boldsymbol{x}_{\boldsymbol{it}}^{\mathbf{'}}\boldsymbol{\beta}_{\mathbf{0}} + \boldsymbol{x}_{\boldsymbol{it}}^{\mathbf{'}}{\mathbf{(}\boldsymbol{\beta}}_{\mathbf{1}}-\boldsymbol{\beta}_{\mathbf{0}})p +\sum_{j=1}^{\vartheta} \varphi_{j}p^{j}$, (20)

where *K*(*p*) is approximated by a polynomial in *p* of chosen degree *ϑ*. Here, the MTE is computed as the partial derivative of the conditional expectation of $y_{it}$with respect to $P\left( z_{it}, \boldsymbol{x}_{\boldsymbol{it}} \right)$:

$MTE\left( \boldsymbol{x}_{\boldsymbol{it}}, P\left( z_{it}, \boldsymbol{x}_{\boldsymbol{it}} \right)\mathbf{=}p \right)\mathbf{=}\frac{\partial E\{y_{it}|\boldsymbol{x}_{\boldsymbol{it}}, P\left( z_{it}, \boldsymbol{x}_{\boldsymbol{it}} \right)\}}{\partial p}= \boldsymbol{x}_{\boldsymbol{it}}^{\mathbf{'}}{\mathbf{(}\boldsymbol{\beta}}_{\mathbf{1}}-\boldsymbol{\beta}_{\mathbf{0}}) +\frac{\partial K(p)}{\partial p}$. (21)

The parameters are estimated by the linear regression implied by equation (20).
The MTE can also be estimated using a semiparametric estimation strategy. In this case, identification depends crucially on the common support assumption for the propensity score. This requires positive frequencies of $\hat{P}\left( z_{it}, \boldsymbol{x}_{\boldsymbol{it}} \right)$in the range of (0, 1) for individuals that do (*d_it_* = 1) or do not (*d_it_* = 0) opt for a voluntary deductible. The semiparametric estimators of the MTE are computed according to equation (21), where, without any further assumptions on *K(p),* the estimation of the last term requires the use of nonparametric techniques for local derivatives. To estimate equation (21), we run the linear regression implied in equation (20) to obtain $\boldsymbol{\beta}_{\boldsymbol{0}}$ and $\boldsymbol{(\beta}_{\boldsymbol{1}}-\boldsymbol{\beta}_{\boldsymbol{0}})$*.* The remaining parameters of the MTE are then obtained from a local polynomial regression (Fan, Gijbels, Hu, & Huang, [1996](#B21)),

$\tilde{y} = y_{it} - \boldsymbol{x}_{\boldsymbol{it}}^{'}{\hat{\boldsymbol{\beta}}}_{\mathbf{0}}- \boldsymbol{x}_{\boldsymbol{it}}^{'}\hat{{(\boldsymbol{\beta}}_{\mathbf{1}}-\boldsymbol{\beta}_{\mathbf{0}}})P(z_{it}, \boldsymbol{x}_{\boldsymbol{it}})$,

on the common support of *P*($z_{it}, \boldsymbol{x}_{\boldsymbol{it}}$) to arrive at an estimate of $\frac{\partial K(p)}{\partial p}$.

Appendix S6: Effect of covariates on health care utilization and moral hazard of having a voluntary deductible: parametric normal specification

|  | (1) | (2) | (3) | (4) | (5) | (6) | (7) | (8) |
| --- | --- | --- | --- | --- | --- | --- | --- | --- |
| Variables | Specialist visits | | GP visits | | Mental health care visits | | Days in the HOSPITAL | |
|  | Treated | Untreated | Treated | Untreated | Treated | Untreated | Treated | Untreated |
|  |  |  |  |  |  |  |  |  |
| Risk aversion | −0.007 (0.021) | −0.012 (0.011) | 0.005 (0.013) | −0.002 (0.006) | 0.078* (0.044) | 0.030 (0.025) | 0.044 (0.082) | 0.027 (0.028) |
| Male | **−0.158** (0.074)** | **−0.030 (0.029)** | −0.186*** (0.041) | −0.134*** (0.022) | −0.107 (0.169) | −0.027 (0.098) | −0.024 (0.172) | 0.051 (0.067) |
| Age | −0.014 (0.015) | −0.009** (0.004) | 0.001 (0.008) | −0.005 (0.004) | 0.037 (0.049) | 0.017 (0.013) | −0.038 (0.026) | −0.003 (0.013) |
| Age^2^ | 0.000 (0.000) | 0.000* (0.000) | 0.000 (0.000) | 0.000** (0.000) | −0.000 (0.001) | −0.000** (0.000) | 0.000* (0.000) | 0.000 (0.000) |
| Employed | −0.012 (0.100) | −0.018 (0.041) | **0.059 (0.044)** | **−0.066** (0.029)** | 0.022 (0.223) | −0.018 (0.118) | −0.494* (0.268) | −0.314*** (0.121) |
| Educ.mid | 0.026 (0.102) | −0.111*** (0.038) | −0.030 (0.057) | −0.049* (0.028) | 0.093 (0.240) | −0.047 (0.133) | −0.190 (0.192) | −0.122 (0.075) |
| Educ.high | −0.020 (0.090) | −0.041 (0.039) | 0.012 (0.060) | −0.059* (0.034) | 0.108 (0.253) | 0.200 (0.136) | −0.545** (0.242) | −0.211*** (0.072) |
| Married | 0.090 (0.093) | −0.019 (0.039) | **0.092 (0.056)** | **−0.038 (0.028)** | **0.799******* (0.204)** | **−0.069 (0.128)** | 0.033 (0.195) | −0.164** (0.072) |
| Good health | −0.162 (0.100) | −0.126*** (0.036) | **−0.056 (0.055)** | **−0.147******* (0.021)** | 0.195 (0.228) | −0.204* (0.123) | −0.133 (0.185) | −0.060 (0.072) |
| MHI-5 | −0.005** (0.003) | −0.006*** (0.001) | −0.010*** (0.002) | −0.007*** (0.001) | −0.005 (0.007) | −0.009*** (0.002) | −0.009 (0.007) | −0.001 (0.002) |
| Chronic cond. | 0.217** (0.099) | 0.208*** (0.036) | 0.153*** (0.050) | 0.201*** (0.027) | 0.146 (0.198) | −0.107 (0.096) | 0.341* (0.192) | 0.261*** (0.073) |
| Smokes | −0.081 (0.075) | 0.042 (0.034) | −0.011 (0.056) | −0.055 (0.034) | 0.212 (0.202) | 0.047 (0.099) | 0.007 (0.181) | 0.055 (0.078) |
| Log-income | −0.158** (0.071) | −0.066** (0.029) | **−0.192******* (0.047)** | **−0.054** (0.023)** | **−0.618** (0.250)** | **−0.084 (0.112)** | 0.133 (0.239) | 0.062 (0.090) |
| K | −0.031 (0.286) | −0.870*** (0.233) | −0.316** (0.151) | −0.393** (0.181) | −0.030 (0.468) | −0.286 (0.385) | 1.545* (0.871) | 1.486** (0.756) |
| rho1–rho0 | 0.839** (0.377) | | 0.077 (0.205) | | 0.256 (0.530) | | 0.058 (1.257) | |
| E(Y1–Y0)@X | −0.240 (0.482) | | −0.615** (0.262) | | −0.306 (0.724) | | 2.896** (1.474) | |
| Constant | 2.645*** (0.841) | 2.262*** (0.292) | 2.314*** (0.455) | 1.981*** (0.232) | 4.383*** (1.559) | 2.655*** (0.874) | 3.801** (1.564) | 0.136 (0.957) |
| Observations | 5,712 | | 9,783 | | 856 | | 1,506 | |

Notes: Bootstrapped standard errors clustered at the household level are presented in parentheses. The coefficients that differ between the treated and untreated are statistically significantly at the 10% level (denoted in **bold**). The specification include wave dummies.

Appendix S8: Effect of covariates on health care utilization and moral hazard of having a voluntary deductible: semiparametric polynomial specification

|  | (1) | | (2) | (3) | (4) | (5) | (6) | (7) | (8) |
| --- | --- | --- | --- | --- | --- | --- | --- | --- | --- |
|  | Specialist visits | | | GP visits | | Mental health care visits | | Days in the hospital | |
| Variables | β_0_ | | (β_1_–β_0_) | β_0_ | (β_1_–β_0_) | β_0_ | (β_1_–β_0_) | β_0_ | (β_1_–β_0_) |
|  |  | |  |  |  |  |  |  |  |
| Risk aversion | −0.009 (0.021) | | −0.028 (0.122) | −0.012 (0.015) | 0.039 (0.083) | 0.090** (0.042) | −0.221 (0.181) | 0.099* (0.056) | −0.519 (0.383) |
| Male | 0.003 (0.061) | | −0.383 (0.361) | −0.159*** (0.042) | 0.155 (0.235) | 0.147 (0.158) | −0.973 (0.764) | 0.218* (0.118) | −1.404* (0.854) |
| Age | −0.006 (0.013) | | −0.018 (0.083) | 0.000 (0.009) | −0.008 (0.058) | 0.002 (0.021) | 0.144 (0.113) | −0.030 (0.020) | 0.239* (0.129) |
| Age^2^ | 0.000 (0.000) | | 0.000 (0.001) | 0.000 (0.000) | 0.000 (0.001) | −0.000 (0.000) | −0.001 (0.001) | 0.000** (0.000) | −0.003* (0.001) |
| Employed | −0.037 (0.098) | | 0.033 (0.666) | −0.002 (0.065) | −0.271 (0.402) | 0.273 (0.195) | −1.657* (0.885) | −0.528** (0.212) | 1.103 (1.818) |
| Educ.mid | −0.002 (0.084) | | −0.839 (0.679) | −0.009 (0.063) | −0.289 (0.434) | −0.345* (0.193) | 1.506* (0.805) | −0.082 (0.144) | −0.542 (1.133) |
| Educ.high | −0.020 (0.091) | | −0.244 (0.647) | 0.054 (0.089) | −0.532 (0.478) | −0.043 (0.201) | 1.021 (0.845) | −0.481*** (0.152) | 1.454 (1.099) |
| Married | 0.037 (0.101) | | −0.356 (0.641) | −0.085 (0.062) | 0.373 (0.348) | −0.264 (0.187) | 1.603** (0.801) | −0.234 (0.149) | 0.701 (0.980) |
| Good health | −0.188** (0.086) | | 0.330 (0.618) | −0.174*** (0.061) | 0.267 (0.294) | −0.727*** (0.195) | 2.707*** (0.895) | −0.115 (0.153) | 0.279 (1.062) |
| MHI-5 | −0.009*** (0.002) | | 0.030* (0.015) | −0.005*** (0.002) | −0.014 (0.009) | −0.017*** (0.004) | 0.046** (0.019) | −0.002 (0.004) | −0.003 (0.032) |
| Chronic cond. | 0.292*** (0.086) | | −0.633 (0.615) | 0.240*** (0.056) | −0.581* (0.336) | −0.152 (0.157) | 0.493 (0.744) | 0.424** (0.176) | −1.028 (1.257) |
| Smokes | −0.149* (0.086) | | 1.258** (0.515) | −0.092 (0.058) | 0.328 (0.292) | −0.006 (0.205) | 0.511 (0.801) | 0.176 (0.122) | −1.121 (0.945) |
| Log-income | −0.108 (0.066) | | 0.280 (0.430) | −0.108** (0.054) | 0.215 (0.246) | −0.084 (0.188) | −0.267 (0.805) | 0.200 (0.153) | −0.970 (1.174) |
| p1 | |  | −3.270 (5.882) |  | −3.672 (3.324) |  | −4.966 (7.540) |  | 15.872 (14.642) |
| p2 | |  | −1.659 (15.335) |  | 8.507 (11.103) |  | 1.911 (11.035) |  | −32.470 (39.748) |
| p3 | |  | −4.607 (19.648) |  | −8.703 (12.885) |  | −2.450 (11.397) |  | 22.289 (53.539) |
| Joint test p1, p2, p3 *p*-value | | | 0.3149 |  | 0.7478 |  | 0.8736 |  | 0.3306 |
| E(Y1–Y0)@X |  | | −1.566** (0.628) |  | −0.960** (0.475) |  | −0.848 (0.906) |  | 0.695 (2.346) |
| Constant | 2.532*** (0.746) | |  | 2.353*** (0.494) |  | 3.340** (1.532) |  | −1.136 (1.716) |  |
| Observations | 5,712 | | 5,712 | 9,783 | 9,783 | 856 | 856 | 1,506 | 1,506 |
