# Supplementary material for: Moral hazard and selection for voluntary deductibles
Source: Health Econ. 2020 Jul 31;29(10):1251–69. doi: 10.1002/hec.4134 (PMC7539990; doi:10.1002/hec.4134)
Supplement: Supplementary file 2 — Data S2. Supporting Information [file HEC-29-1251-s002.zip › Appendix_S7.docx]

Appendix S7: MTEs
